# Prognostic value of baseline ^18^F-FDG PET/CT metabolic parameters in EGFR-mutant lung adenocarcinoma patients treated with EGFR-TKIs

**DOI:** 10.3389/fnume.2026.1864776

**Published:** 2026-07-08

**Authors:** Lei Wang, Zhenpeng Wang, Wei Wu

**Affiliations:** 1 Department of Nuclear Medicine, Jilin Cancer Hospital, Changchun, China; 2 Department of Internal Medicine, Liaoyuan Central Hospital, Liaoyuan, China

**Keywords:** ^18^F-FDG PET/CT, 19del, EGFR-mutant lung adenocarcinoma, L858R, metabolic parameters, prognosis, tyrosine kinase inhibitors

## Abstract

**Objective:**

To investigate the correlation between baseline ^1^⁸F-FDG PET/CT metabolic parameters and progression-free survival (PFS) in EGFR-mutant lung adenocarcinoma receiving EGFR-TKI treatment, with subgroup analysis for exon 19 deletion (19del) and L858R mutations.

**Methods:**

This single-center retrospective study included 175 patients with pathologically confirmed EGFR-mutant lung adenocarcinoma who underwent baseline ^1^⁸F-FDG PET/CT prior to EGFR-TKI therapy between 2020 and 2023. Quantitative metabolic parameters, including SUVmax, SUVpeak, metabolic tumor volume (MTV), and total lesion glycolysis (TLG), were measured. Progression-free survival was the primary endpoint. Survival analyses were performed using Kaplan–Meier estimates, log-rank tests, and Cox proportional hazards regression models, with prespecified subgroup analyses according to EGFR mutation subtype (19del vs. L858R).

**Results:**

The median follow-up duration was 48 months. In univariate analysis, higher SUVmax, SUVpeak, MTV, and TLG were significantly associated with shorter PFS (all *P* < .05), while MTV and TLG failed to reach statistical significanceOn multivariate Cox regression analysis, SUVmax remained independently associated with PFS [hazard ratio (HR) = 1.031, 95% confidence interval 1.001–1.059, *P* = .045]. Patients with EGFR 19del mutations demonstrated a significantly longer median PFS compared with those harboring L858R mutations (13.57 vs. 10.37 months, *P* = .02). Third-generation EGFR-TKIs were associated with a significant prolongation of PFS in the 19del subgroup (*P* < .05), whereas no significant benefit was observed in the L858R subgroup, these subgroup findings should be interpreted cautiously as preliminary exploratory observations limited by small sample sizes.

**Conclusion:**

Baseline ^1^⁸F-FDG PET/CT metabolic parameters, especially SUVmax, are independent prognostic biomarkers for PFS in EGFR-mutant lung adenocarcinoma patients receiving EGFR-TKIs therapy. The prognostic implications of metabolic imaging differ according to EGFR mutation subtype, and further large prospective cohorts are needed to validate our exploratory subgroup findings supporting an integrated approach combining metabolic imaging and molecular stratification for individualized risk assessment.

## Introduction

Lung cancer remains the leading cause of cancer-related mortality worldwide, with non-small cell lung cancer (NSCLC) accounting for approximately 85% of all cases (1). Lung adenocarcinoma is the most prevalent histological subtype of NSCLC and is frequently associated with driver gene alterations, among which epidermal growth factor receptor (EGFR) mutations are the most common, particularly in East Asian populations (2). The introduction of EGFR tyrosine kinase inhibitors (TKIs) has fundamentally changed the therapeutic landscape of EGFR-mutant lung adenocarcinoma and significantly prolonged patient survival (3, 4). Nevertheless, substantial heterogeneity in treatment response and progression-free survival (PFS) persists, and both primary and acquired resistance remain major clinical challenges.

^18^F-fluorodeoxyglucose positron emission tomography/computed tomography (^18^F-FDG PET/CT) enables non-invasive quantification of tumor glucose metabolism and has become an indispensable tool for tumor staging, treatment response assessment, and prognostic evaluation in lung cancer (5). Metabolic parameters derived from PET/CT, including intensity-based indices such as the maximum standardized uptake value (SUVmax) and volumetric parameters such as metabolic tumor volume (MTV) and total lesion glycolysis (TLG), have been proposed as imaging biomarkers reflecting tumor burden, biological aggressiveness, and intratumoral heterogeneity (6).

Previous studies have suggested that EGFR-mutant tumors generally exhibit lower FDG uptake than wild-type tumors, and baseline PET/CT metabolic parameters may correlate with survival outcomes in patients receiving EGFR-TKI therapy (7). However, several important limitations remain in the existing literature. First, most studies analyzed EGFR-mutant lung adenocarcinoma as a single entity, without adequately addressing the biological and clinical differences between the two most prevalent EGFR mutation subtypes—exon 19 deletion (19del) and L858R substitution (8). Second, many previous cohorts were treated predominantly with first- or second-generation TKIs (9), limiting applicability to contemporary clinical practice in which third-generation TKIs are increasingly used as first-line therapy. Third, the relative prognostic value of volumetric metabolic parameters vs. intensity-based metrics such as SUVmax remains controversial, particularly in genetically defined subgroups (10).

To address these gaps, this study aimed to systematically evaluate the prognostic and predictive value of baseline ^18^F-FDG PET/CT metabolic parameters in a contemporary cohort of EGFR-mutant lung adenocarcinoma patients treated with EGFR-TKIs. Furthermore, we performed prespecified subgroup analyses according to EGFR mutation subtype (19del vs. L858R) to explore potential differences in metabolic imaging prognostic performance and treatment benefit, thereby providing imaging-based evidence to support individualized risk stratification and therapeutic decision-making.

## Methods

### Study design and patient population

This retrospective study was approved by the Ethics Committee of Jilin Cancer Hospital (No.20211031-01). Informed consent was waived due to the retrospective design. Patients with pathologically confirmed EGFR-mutant lung adenocarcinoma between January 2020 and December 2023 were screened.

### Inclusion criteria

1. Pathologically confirmed EGFR-mutant lung adenocarcinoma;
2. Baseline ^18^F-FDG PET/CT performed within 30 days before treatment;
3. Complete clinical and follow-up data;
4. Clinical stage IIIA–IV or inoperable stage I–II;
5. Fasting blood glucose 4–11 mmol/L.

### Exclusion criteria

1. History of other malignant tumors;
2. Prior antineoplastic therapy before PET/CT or genetic testing;
3. Treatment discontinuation due to withdrawal or adverse events;
4. Metastatic lesions without pathological confirmation.

### Treatment regimens

Patients received first-, second-, or third-generation EGFR-TKIs until disease progression or intolerable toxicity. Subgroups: 19del, L858R, and other rare EGFR mutations.

### Treatment regimens

Patients received first-, second-, or third-generation EGFR-TKIs until disease progression or intolerable toxicity. Specific administered agents: first-generation (gefitinib, erlotinib, icotinib); second-generation (afatinib, dacomitinib); third-generation (osimertinib, almonertinib, furmonertinib). We recorded the distribution of first-line vs. subsequent-line TKI administration, treatment switching events and detailed post-progression salvage therapies after disease progression. Subjects were stratified into 19del, L858R, and other rare EGFR mutation subgroups for subsequent analyses.

### Imaging protocol

Scans were acquired on a GE Discovery PET/CT 710 system. Patients fasted ≥6 h; 3.7–5.5 MBq/kg ^18^F-FDG was injected intravenously. Imaging started 50–60 min post-injection. CT: 120 kV, 300 mA, pitch 0.705, 1-mm slice thickness. PET: 3D mode, 1 min per bed position.

### Image analysis

Two experienced nuclear medicine physicians independently drew primary regions of interest (ROI). MTV and TLG were calculated using a fixed threshold of SUV ≥2.5, which was adopted to ensure reproducibility and facilitate comparison with previous lung cancer PET studies. Notably, this absolute threshold may underestimate tumor burden in subsets of EGFR-mutant lesions with inherently low FDG avidity.Discrepancies were resolved by consensus. Interobserver agreement was assessed using ICC; mean values were used for analysis.

### Follow-up and endpoint definition

Patients were followed up every 6–8 weeks. PFS was defined as the interval from treatment initiation to disease progression per RECIST 1.1 criteria or death from any cause. The primary endpoint was PFS. Overall survival was not selected as the primary endpoint due to the relatively high rate of treatment crossover and insufficient death events during follow-up.

### Statistical analysis

SPSS 22.0 and MedCalc 19.0 were used. Continuous variables were compared by t-test or Mann–Whitney U test; categorical variables by *χ*^2^ test. ROC analysis determined optimal cutoffs. Survival curves were constructed by Kaplan–Meier and compared by log-rank test. Univariate and multivariate Cox regression were applied to screen independent prognostic factors, with strict standardized model construction rules as below:

1. Variable entry criterion: All variables with P＜.10 in univariate Cox analysis plus clinically meaningful baseline parameters were enrolled into multivariate regression;
2. Proportional hazards assumption: Schoenfeld residual test was conducted; all enrolled variables satisfied proportional hazards assumption (all P＞.05);
3. Collinearity assessment: Variance inflation factor (VIF) analysis for PET metabolic indicators; no severe multicollinearity was identified (all VIF＜5).
4. *P* < .05 was considered statistically significant.

## Results

### Patient baseline characteristics

A total of 175 patients were included in this study, comprising 77 males and 98 females, with a mean age of 60.52 years. Most patients had advanced disease at diagnosis, with 155 patients (88.6%) classified as stage IV. Regarding EGFR mutation status, 89 patients (50.9%) harbored the L858R mutation, 65 patients (37.1%) had exon 19 deletion (19del), and 21 patients (12.0%) had other less common EGFR mutations. Detailed baseline clinical characteristics and TKI treatment classification data are summarized in Table 1.

**Table 1:** Baseline clinical characteristics of 175 patients with EGFR-mutant lung adenocarcinoma.

Characteristics	No. %
Sex	
Male	77 (44.0)
Female	98 (56.0)
Age	
≤65	115 (65.7)
>65	60 (34.3)
EGFR mutations type	
L858R	89 (50.9)
19Del	65 (37.1)
Others	21 (12.0)
Smoking	
Never	146 (83.4)
Current/Former	29 (16.6)
TNM Stage	
II	1 (0.6)
II	19 (10.9)
IV	155 (88.6)
TKI generation	
1st-generation	72 (41.1)
2nd-generation	16 (9.1)
3rd-generation	87 (49.7)
Post-progression switched therapy	43(24.6)

### Baseline metabolic parameters

The ICC values for SUVmax, SUVpeak, MTV, and TLG were 0.93, 0.91, 0.88, and 0.86, respectively, indicating good to excellent interobserver agreement. Therefore, the averaged measurements were considered reliable for subsequent survival analyses. The median baseline values of PET-derived metabolic parameters were as follows: SUVmax 10.59, SUVpeak 8.29, MTV 13.33 cm^3^, and TLG 73.58. No statistically significant differences in SUVmax, SUVpeak, MTV, or TLG were observed between patients with 19del and those with L858R mutations (all *P* > .05; Table 2).

**Table 2:** Clinical and imaging characteristics according to EGFR mutations subtype.

Characteristics	19Del(*n* = 65)	L858R(*n* = 89)	*P*-value
Age,y,(range)	57.64 (51–66)	61.90 (54–68)	＞.05
Age≤65 y	46 (70.8)	55 (61.8)	＞.05
Female sex	29 (44.6)	57 (64.0)	＜.05
Never smoking	55 (84.6)	74 (83.1)	＞.05
TNM stage			＞.05
II	1 (1.5)	0 (0.0)	
III	7 (10.8)	8 (9.0)	
IV CT features	57 (87.7)		
Smooth margin	3 (4.6)	10 (11.2)	＞.05
Vacuole Sign	9 (13.8)	13 (14.6)	＞.05
Spiculation	37 (56.9)	44 (49.4)	＞.05
Vascular convergence	30 (46.2)	36 (40.4)	＞.05
Lobulation	53 (81.5)	65 (73.0)	＞.05
Pleural indentation	38 (58.5)	62 (69.7)	>.05
Metastases			
Brain	16 (24.6)	25 (28.1)	＞.05
Liver	12 (18.5)	6 (6.7)	＜.05
Adrenal	3 (4.6)	10 (11.2)	＞.05
Bone	34 (52.3)	44 (49.4)	＞.05
Pleural	26(40.0)	33(37.1)	＞.05

Data are presented as No. (%) unless otherwise indicated.

### ROC analysis for PFS stratification

Receiver operating characteristic (ROC) analysis was performed to explore potential reference cutoff values of baseline PET-derived metabolic parameters for progression-free survival (PFS). The area under the curve (AUC) values were modest, with an AUC of 0.582 for SUVmax (optimal cutoff: 11.48), 0.579 for SUVpeak (cutoff: 6.99), and 0.581 for TLG (cutoff: 38.19), indicating limited discriminatory performance when these parameters were considered individually.

Given the time-to-event nature of PFS and the continuous distribution of metabolic variables, ROC analyses were regarded as exploratory. The identified cutoff values were therefore applied primarily for descriptive Kaplan–Meier survival analyses rather than for definitive outcome prediction.

### Survival analysis

In univariate Cox regression analysis, age > 65 years, EGFR exon 19 deletion, SUVmax, SUVmin, SUVavg, and SUVpeak were significantly associated with PFS (all *P* < .05). In multivariate Cox proportional hazards analysis, SUVmax remained independently associated with PFS [hazard ratio (HR) = 1.031, per 1-unit increase in continuous SUVmax,95% confidence interval 1.001–1.059, *P* = 0.045], after adjustment for relevant clinical and imaging variables (Table 3).

**Table 3:** Univariate and multivariate Cox regression analyses for the progression-free survival.

Characteristics	Univariate analysis		Multivariate analysis	
	HR(95%CI)	*p*-value	HR(95%CI)	*P*-value
Age≥65y	1.542 (1.072–2.217)	.020*	1.449 (1.009–2.081)	.371
Female sex	1.280 (0.904–1.813)	.164		
Current/former Smoking	1.239 (0.784–1.960)	.359		
TNM Stage(IVvs II/ III)	0.656 (0.091–4.725)	.676		
EGFR mutations (19Del vs. L858R)	0.647 (0.444–0.943)	.023*		
SUV max	1.030 (1.004–1.058)	.024*	1.031 (1.001–1.059)	.045*
SUV min	1.085 (1.003–1.174)	.042*		
SUV avg	1.056 (1.007–1.108)	.025*		
SUV peak	1.037 (1.002–1.072)	.036*		
TLG	1.000 (1.000–1.001)	.129		
MTV	1.001 (0.998–1.005)	.437		
Brain metastases	1.302 (0.889–1.907)	.176		
liver metastases	1.258 (0.709–2.232)	.433		
Adrena metastasis	1.301 (0.967–1.752)	.082		
Bone metastasis	0.810 (0.577–1.138)	.224		
Pleural metastasis	1.088 (0.764–1.551)	.640		

HR, hazard ratio; CI, confidence interval; SUV_max_, maximum standardized uptake value; MTV, metabolic tumor volume; TLG, total lesion glycolysis. All metabolic parameters were analyzed as continuous variables in Cox regression models. HR represents the hazard ratio per 1-unit increment of each parameter. ROC-derived cut-off values were only used for Kaplan–Meier survival analyses.

**Figure 1:**
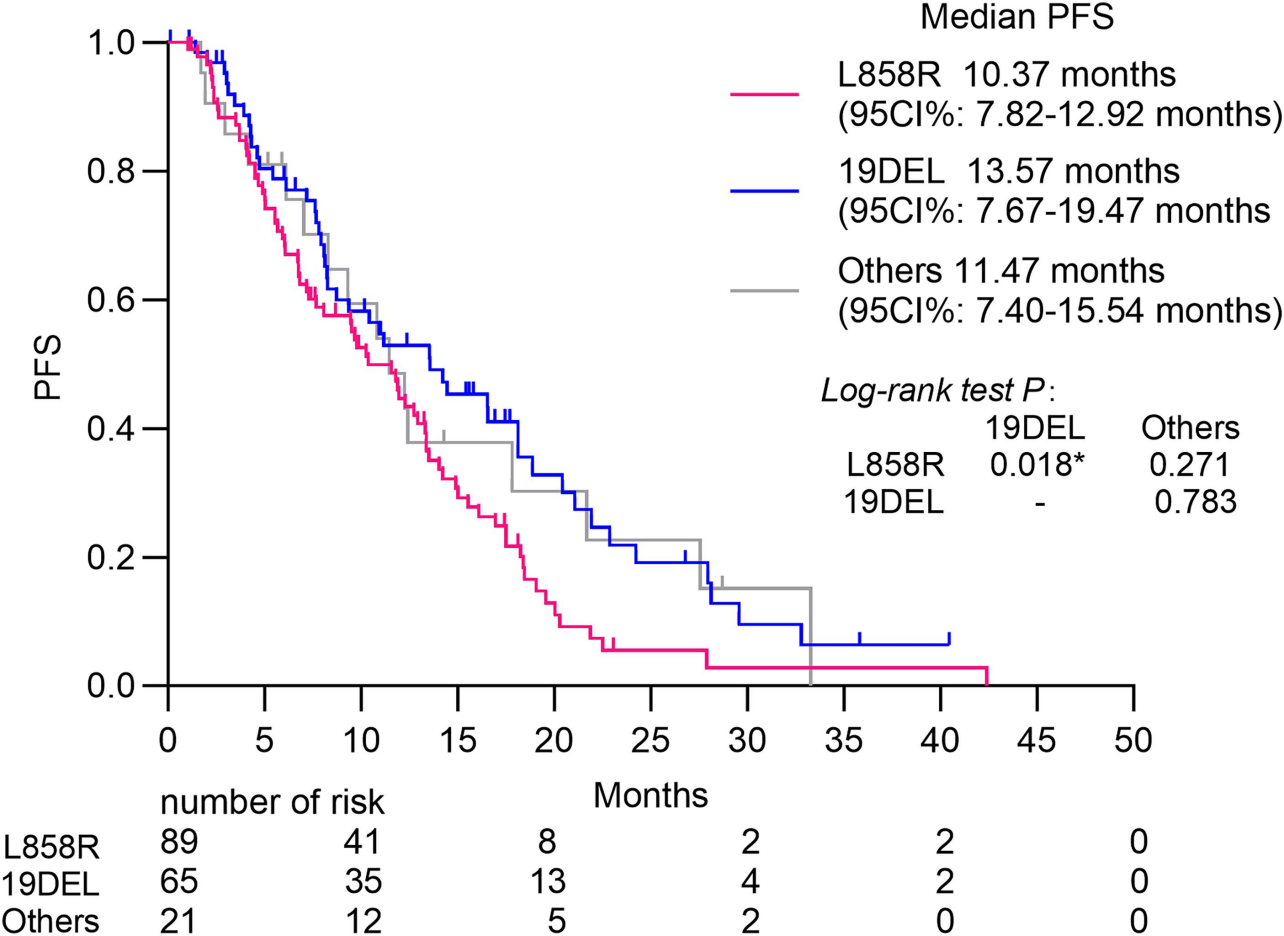
Kaplan–meier curves of progression-free survival (PFS) according to EGFR mutation subtype in patients with EGFR-mutant lung adenocarcinoma treated with EGFR-tyrosine kinase inhibitors.

**Figure 2:**
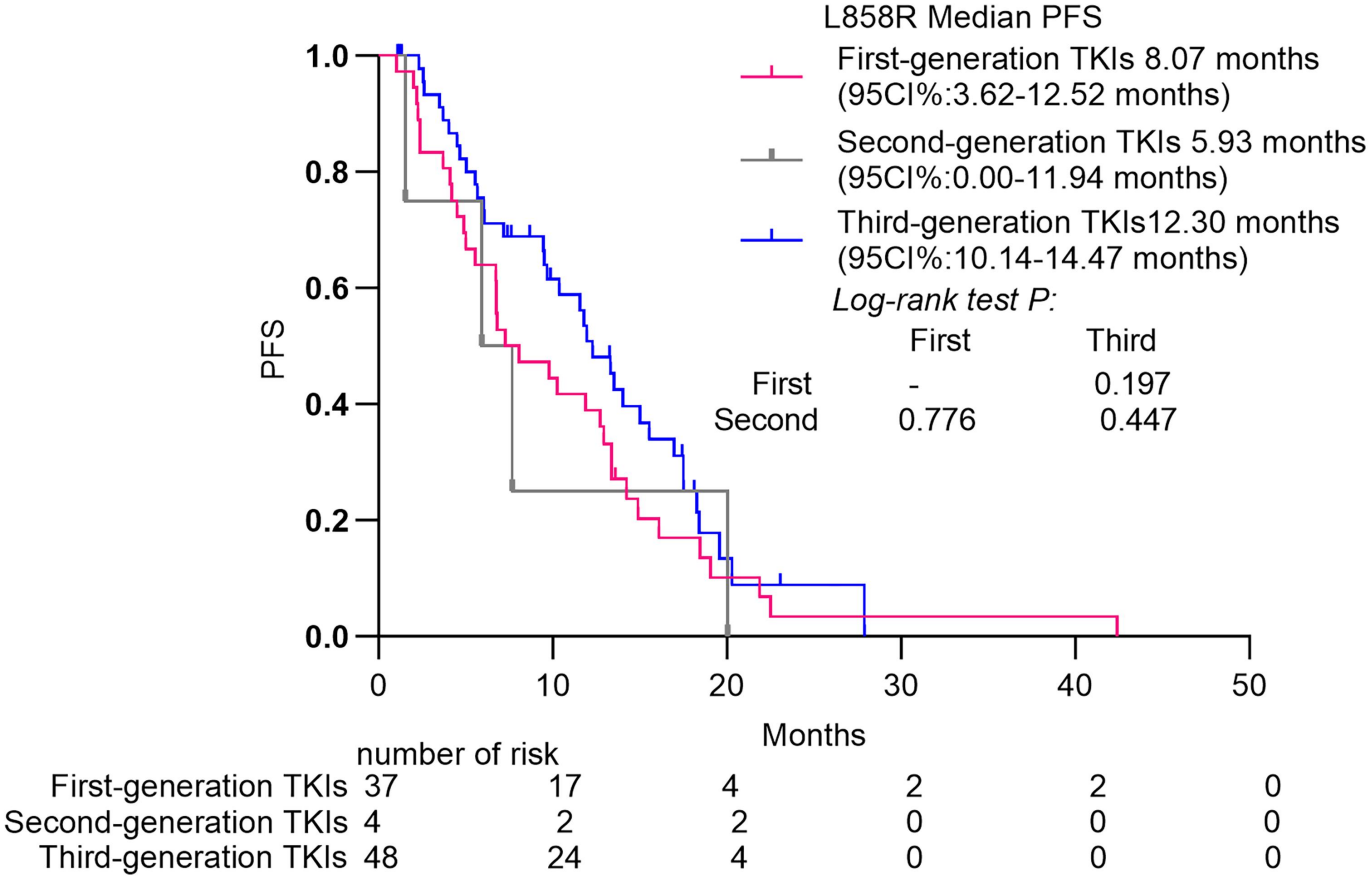
Comparison of PFS with first-, second-, and third-generation EGFR-tyrosine kinase inhibitors in patients harboring the L858R mutation.

**Figure 3:**
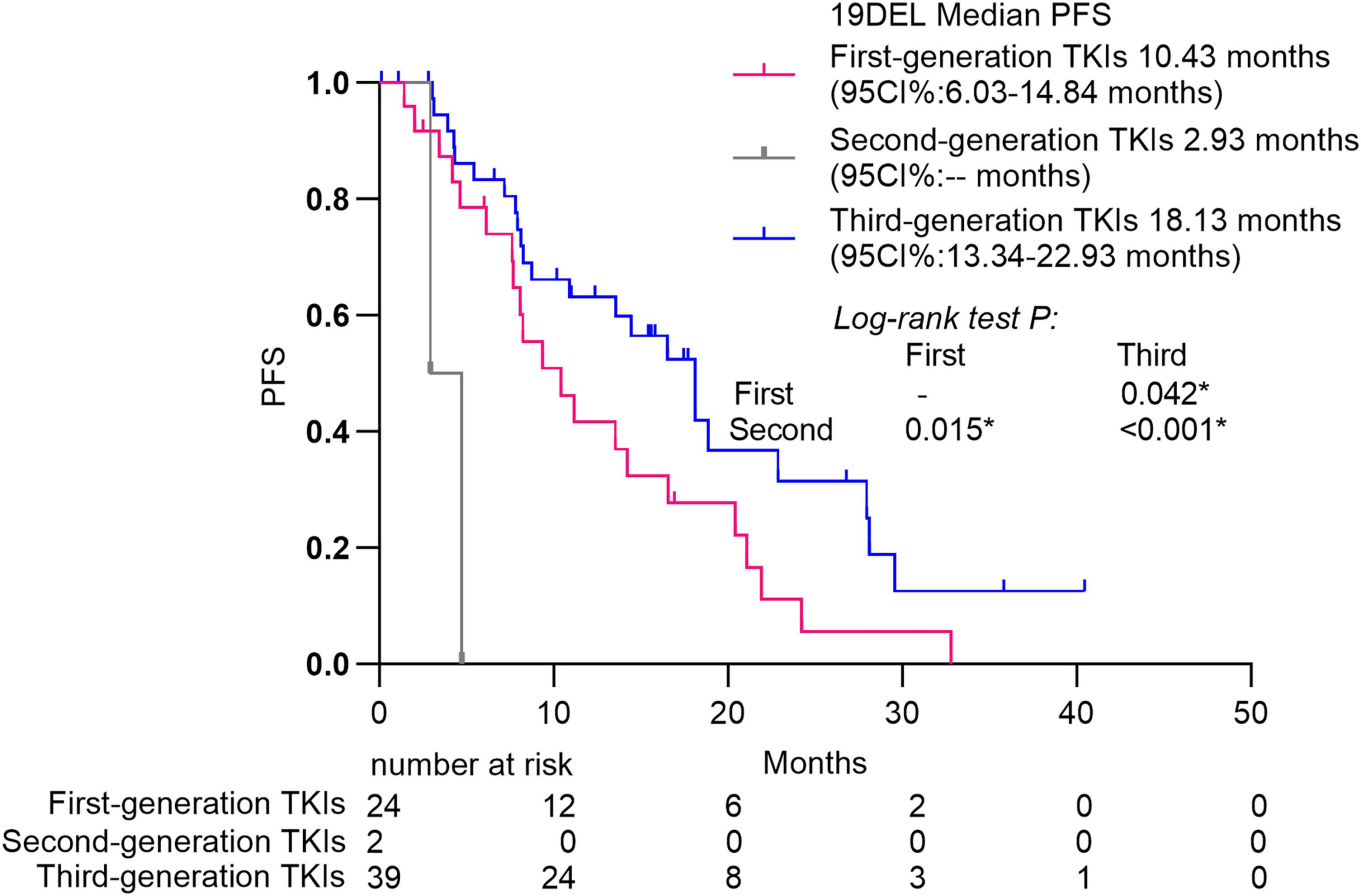
Comparison of PFS with first-, second-, and third-generation EGFR-tyrosine kinase inhibitors in patients harboring the 19Del mutation.

Patients with EGFR 19del mutations exhibited a significantly longer median PFS compared with those harboring L858R mutations (13.57 months vs. 10.37 months, *P* = .02). Subgroup analysis according to TKI generation demonstrated that third-generation EGFR-TKIs were associated with a significant prolongation of PFS in the 19del subgroup (*P* < .05), whereas no statistically significant benefit was observed in the L858R subgroup (*P* = 0.197)As explicitly noted in Discussion, several subgroups suffered extremely limited sample size: *n* = 2 for 19del + second-generation TKI, *n* = 4 for L858R + second-generation TKI, leading to insufficient statistical power; patients with rare EGFR mutations (*n* = 21) were excluded from intersubgroup comparative analyses and were not included in survival curves (Figures 1–3).

### Representative imaging cases

**Figure 4:**
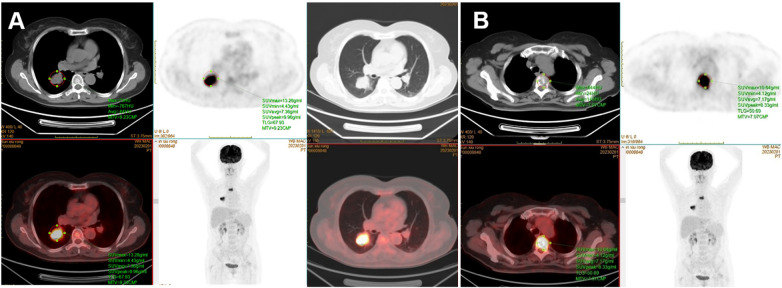
Representative ^18^F-FDG PET/CT images of a 66-year-old woman with EGFR 19Del-positive lung adenocarcinoma (cT4N0M1c, stage ⅣB). **(A)** Primary tumor in the right lower lobe: SUV_max_=13.28, SUV_min_=4.43, SUV_avg_=7.36, SUV_peak_=9.96, TLG=67.93, MTV=9.23 cm^3^. **(B)** Sternal metastasis: SUV_max_=10.64, SUV_min_=4.12, SUV_avg_=7.17, SUV_peak_=8.33, TLG=50.69, MTV=7.07 cm^3^.

**Figure 5:**
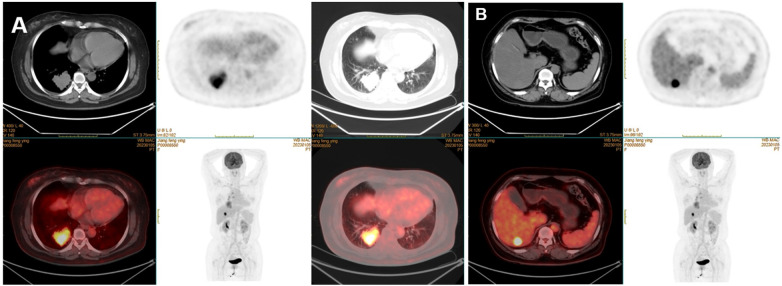
Representative ^18^F-FDG PET/CT images of a 54-year-old woman with EGFR L858R-positive lung adenocarcinoma (cT4N0M1c, stage ⅣB). **(A)** Primary tumor in the right lower lobe: SUV_max_=8.53, SUV_min_=3.34, SUV_avg_=4.77, SUV_peak_=7.52, TLG=240.46, MTV=50.41 cm^3^. **(B)** Hepatic metastasis: SUV_max_=13.66, SUV_min_=4.89, SUV_avg_=8.04, SUV_peak_=11.28, TLG=46.15, MTV=5.74 cm^3^.

Representative baseline ^18^F-FDG PET/CT images of patients with EGFR 19del and L858R mutations, illustrating primary tumors and metastatic lesions, are presented in Figures 4, 5.

## Discussion

Despite the transformative impact of epidermal growth factor receptor tyrosine kinase inhibitors (EGFR-TKIs) on the management of lung adenocarcinoma, clinical outcomes remain heterogeneous among patients harboring activating mutations. This variability underscores an urgent need for robust, non-invasive biomarkers capable of refining risk stratification and optimizing therapeutic algorithms beyond molecular genotyping alone. While next-generation sequencing has elucidated the genomic landscape, including rare fusions and compound mutations that influence prognosis, functional imaging offers a complementary perspective by capturing the metabolic phenotype of the tumor burden (11, 12) Consequently, integrating quantitative metabolic parameters with specific mutational subtypes represents a critical frontier in precision oncology to address the unmet need for predicting treatment response and disease progression in this distinct patient population.

In this retrospective study, we investigated the prognostic utility of baseline ^18^F-FDG PET/CT metabolic parameters within a rigorously characterized cohort of EGFR-mutated lung adenocarcinoma patients treated with TKIs. By leveraging a homogeneous population confirmed through standardized pathological and genetic protocols, our analysis aimed to minimize confounding variables often present in broader cohorts. We identified that elevated baseline maximum standardized uptake value (SUVmax) serves as an independent predictor of shortened progression-free survival, while also confirming the superior outcomes associated with exon 19 deletions compared to L858R point mutations. Furthermore, our data revealed a nuanced interaction between mutation subtypes and TKI generations, suggesting that metabolic activity may differentially modulate therapeutic efficacy across molecular strata. The following discussion interprets these findings in the context of existing literature, exploring the biological underpinnings of metabolic heterogeneity and their implications for personalized treatment strategies. We cannot draw definitive conclusions about superior therapeutic efficacy of third-generation TKIs between 19del and L858R patients, considering single-center retrospective design and inadequate subgroup sample size; all inter-subgroup survival differences are only preliminary exploratory observations, and large-scale prospective multicenter cohorts are required for subsequent external validation.）

Building upon the identification of baseline SUVmax as an independent prognostic factor, our findings underscore the biological significance of tumor glycolytic activity in predicting EGFR-TKI resistance, a phenomenon consistent with the Warburg effect where heightened glucose metabolism reflects aggressive tumor phenotypes and rapid proliferation (13) While previous studies in nasopharyngeal carcinoma and renal cell carcinoma have established SUVmax as a robust predictor of survival across diverse malignancies, our data specifically validate this metric within the homogenous context of EGFR-mutant lung adenocarcinoma, where high metabolic flux may indicate activated PI3 K/AKT/mTOR signaling pathways that bypass TKI inhibition (14, 15) The limited discriminative power of individual metabolic parameters observed in our ROC analysis, contrasting with their strong performance in univariate survival models, likely stems from the relatively uniform molecular background of our cohort; unlike heterogeneous non-small cell lung cancer populations where metabolic variance is driven by distinct driver mutations, EGFR-mutant tumors share common downstream metabolic reprogramming, thereby compressing the dynamic range of SUV values needed for binary classification (16) Furthermore, the superior inter-observer reproducibility of SUVmax compared to volumetric parameters like MTV or TLG reinforces its utility as a standardized biomarker, particularly given that fixed-threshold segmentation methods often fail to account for varying background uptake in different scanner protocols, a limitation noted in lymphoma studies where semi-automated contours showed variable prognostic impact (17)Consequently, while SUVmax serves as a reliable continuous risk stratifier reflecting intrinsic tumor aggressiveness, its modest AUC suggests it should be integrated with other molecular markers rather than used in isolation for binary decision-making. As a key limitation of this single-center retrospective research, several stratified subgroups contained extremely small sample sizes (*n* = 2 in 19del + second-generation EGFR-TKI group; *n* = 4 in L858R + second-generation EGFR-TKI group), resulting in unstable statistical estimation and insufficient statistical power. All subgroup-related outcomes in our study should be cautiously interpreted due to uneven subgroup distribution and scarce case number, and these findings cannot be directly extrapolated to clinical practice.）

The pronounced survival disparity between patients harboring exon 19 deletions (19del) and those with L858R mutations observed in our cohort aligns with emerging proteomic evidence suggesting these genotypes originate from distinct cellular lineages with divergent downstream signaling networks (18)Specifically, L858R mutants have been shown to preferentially activate causal networks involving PARPBP and HOXA1, which may confer a more aggressive phenotype and reduced sensitivity to TKIs compared to the ASGR1 and MAPK10-dominated pathways prevalent in 19del tumors18 This mechanistic divergence is further corroborated by clinical data indicating that concomitant mutations, which are equally prevalent in both subgroups, exert a more detrimental effect on objective response rates in L858R patients, potentially due to synergistic activation of the HGF/c-Met pathway that is uniquely associated with this mutation type (19) Our subgroup analysis revealing that third-generation TKIs significantly prolong progression-free survival only in the 19del cohort, but not in L858R patients, mirrors findings from large-scale retrospective analyses where specific 19del subtypes demonstrated superior outcomes and distinct resistance mechanisms compared to L858R variants (20) Although some studies argue that mutation abundance rather than type drives prognosis, our results support the hypothesis that the structural conformation of the 19del mutant allows for higher binding affinity and more durable inhibition by osimertinib, whereas the L858R configuration may facilitate earlier acquisition of bypass tracks or secondary resistance mutations (21) Thus, the differential efficacy of third-generation TKIs across mutation subtypes necessitates a genotype-specific therapeutic approach rather than a uniform treatment strategy.

The statistical trajectory wherein SUVmax retained independent prognostic value while other metabolic parameters and clinical variables lost significance in multivariate analysis highlights the potential collinearity between tumor burden metrics and peak metabolic intensity, suggesting that SUVmax captures the most biologically active clone driving disease progression (22) This observation is consistent with studies in multiple myeloma where metabolic heterogeneity, specifically the lesion with the highest SUVmax, proved more prognostically relevant than total metabolic tumor volume, implying that the most aggressive subclone dictates overall survival outcomes regardless of total tumor load (22) The failure of age and other SUV derivatives to remain independent predictors may also reflect the overriding influence of molecular drivers in this population; for instance, concurrent TP53 mutations, which are known to worsen prognosis in EGFR-mutant lung cancer, might be more strongly correlated with high SUVmax than with patient age, thereby masking the effect of demographic factors in our model (23) Moreover, the methodological choice to treat SUVmax as a continuous variable in Cox regression avoids the information loss inherent in dichotomization via ROC-derived cutoffs, a practice increasingly criticized for creating artificial risk groups that do not reflect the linear relationship between metabolic activity and hazard ratios seen in lymphoma and head and neck cancer cohorts (24, 25) While ROC analysis provided descriptive thresholds for Kaplan–Meier visualization, the continuous nature of the hazard ratio confirms that even incremental increases in glycolytic activity correspond to elevated risks, supporting the integration of SUVmax as a linear covariate in future predictive nomograms alongside genomic markers.

The absence of significant differences in baseline metabolic parameters between 19del and L858R subgroups, despite their divergent clinical outcomes, suggests that glucose metabolism as measured by FDG-PET may not be the primary phenotypic mediator of the survival disparity driven by these specific EGFR mutations18 This finding implies that the distinct proteomic landscapes and signaling pathway activations characterizing 19del vs. L858R tumors, such as differential regulation of cell cycle and immune response genes, do not necessarily translate into grossly different levels of global glucose avidity detectable by standard PET metrics18 Furthermore, the similar distribution of compound mutations and co-occurring genetic alterations across both subgroups in our cohort supports the notion that metabolic homogeneity exists at the macroscopic imaging level, even when microscopic molecular heterogeneity drives differential drug sensitivity12 This decoupling of metabolic phenotype from mutational genotype contrasts with observations in subsolid nodules where EGFR mutations are associated with lower consolidation-to-tumor ratios and less aggressive radiological features compared to KRAS mutations, indicating that while mutation type influences morphological invasiveness, it may not uniformly dictate metabolic intensity in advanced solid tumors (26) Additionally, the presence of germline EGFR mutations or rare variants like exon 19 insertions, which were excluded or underrepresented in our comparative analysis, might introduce distinct metabolic signatures that differ from common somatic mutations, warranting further investigation in larger, multi-center cohorts to fully elucidate the genotype-metabolism interface (27, 28) Therefore, while metabolic imaging provides crucial prognostic information, it appears to operate orthogonally to the specific mutational subtype in determining therapeutic response, necessitating a multimodal assessment strategy.

The interaction between TKI generation and mutation subtype observed in our study, where third-generation agents conferred a distinct benefit only in 19del patients, underscores the complexity of treatment selection in the era of precision oncology and highlights the limitations of retrospective data in disentangling line-of-therapy biases (29) While prospective trials like FLAURA have established osimertinib as the preferred first-line agent for all sensitizing EGFR mutations, *post-hoc* analyses and real-world evidence suggest that the magnitude of benefit may vary by genotype, with L858R patients potentially deriving less absolute survival gain compared to those with 19del, possibly due to higher rates of *de novo* resistance or distinct bypass mechanisms20 The confounding effect of treatment heterogeneity in our cohort, where patients received various TKI generations based on clinical availability and physician preference rather than randomization, mirrors challenges noted in studies evaluating combination therapies, where the addition of chemotherapy to TKIs showed variable efficacy across mutation subtypes (30) Nevertheless, the trend toward improved outcomes with third-generation TKIs in the 19del group aligns with mechanistic data showing that newer inhibitors effectively target not only sensitizing mutations but also common resistance variants like T790M, which may arise more frequently or earlier in specific mutational contexts (31) Future prospective designs must stratify by both mutation subtype and metabolic risk to determine whether high-SUVmax L858R patients require intensified upfront strategies, such as combination therapy or earlier switching to fourth-generation agents targeting C797S resistance, to overcome their inherently poorer prognosis (32, 33)

This study has several limitations. First, the retrospective, single-center design with a modest sample size (*n* = 175) inherently limits the generalizability of our findings. While the cohort was homogeneous regarding EGFR mutation status, the statistical power for subgroup analyses, particularly for evaluating the differential efficacy of third-generation TKIs within specific mutation subtypes, was constrained. This may have obscured more nuanced interactions between metabolic parameters and treatment outcomes. Second, the heterogeneity in treatment regimens, encompassing different generations of EGFR-TKIs and varying lines of therapy, introduces a potential confounding effect that cannot be fully eliminated by multivariate adjustment. Although we attempted to control for this, residual confounding may influence the observed associations between SUVmax and progression-free survival. Third, we used a fixed absolute threshold of SUV ≥ 2.5 for volumetric parameter measurement. This approach may underestimate MTV and TLG in EGFR-mutant lesions with low FDG avidity. Although relative adaptive thresholds can alleviate this issue, they may cause over-segmentation of peritumoral inflammation and have poor inter-center reproducibility. Further studies are required to identify the optimal segmentation strategy for this patient population.

In summary, this study identifies baseline SUVmax as an independent prognostic factor for PFS in EGFR-mutant lung adenocarcinoma patients treated with EGFR-TKIs and reinforces the prognostic divergence between the 19del and L858R subtypes. The finding that the benefit of third-generation TKIs appears more pronounced in the 19del subgroup adds a novel imaging-based perspective to the existing molecular stratification. These results underscore the potential clinical utility of integrating quantitative metabolic imaging with genotypic data for refined risk assessment. Future prospective, multi-center studies with larger cohorts and standardized treatment protocols are warranted to validate these observations and to explore the construction of integrated prognostic models incorporating SUVmax. Furthermore, investigating the biological underpinnings of high glycolytic activity through correlative multi-omics analyses could elucidate its role in therapeutic resistance and guide the development of novel combination strategies.

## Conclusion

In summary, baseline SUVmax derived from ^18^F-FDG PET/CT was independently associated with progression-free survival in patients with EGFR-mutant lung adenocarcinoma treated with EGFR-TKIs. The prognostic relevance of metabolic parameters appears to be influenced by EGFR mutation subtype and treatment generation. These exploratory findings support a multimodal approach combining metabolic imaging and molecular profiling for pretreatment risk stratification, while further prospective multi-center trials are required to verify our results before clinical popularization.

## Data Availability

The raw data supporting the conclusions of this article will be made available by the authors, without undue reservation.
